# Effect of Inter-Hospital Transfer on Mortality in Patients Admitted through the Emergency Department

**DOI:** 10.3390/jcm13164944

**Published:** 2024-08-22

**Authors:** Jei-Joon Song, Si-Jin Lee, Ju-Hyun Song, Sung-Woo Lee, Su-Jin Kim, Kap-Su Han

**Affiliations:** Emergency Department, College of Medicine, Korea University, 73 Inchon-ro, Seongbuk-gu, Seoul 02841, Republic of Korea; neigh436@gmail.com (J.-J.S.); songcap97@hotmail.com (J.-H.S.); kuedlee@korea.ac.kr (S.-W.L.); icarusksj@korea.ac.kr (S.-J.K.)

**Keywords:** inter-hospital transfer, emergency department, in-hospital mortality

## Abstract

**Background:** Despite advancements in emergency medical systems, inter-hospital transfer (IHT) remains a critical component. Several studies have analyzed the impact of IHT on patient outcomes. Some studies have reported positive effects, indicating that transfers can improve patient prognosis. However, other studies have suggested that transfers may worsen outcomes. We investigated whether IHT is associated with in-hospital mortality. **Methods:** This retrospective observational study utilized data on patient outcomes from the National Emergency Department Information System (NEDIS) from 2016 to 2018, focusing on patients admitted to hospitals after visiting the emergency department (ED). The primary outcome was the in-hospital mortality rate. **Results:** This study included 2,955,476 adult patients admitted to emergency medical centers, with 832,598 (28.2%) undergoing IHT. The in-hospital mortality rate was significantly higher in the transfer group (6.9%) than in the non-transfer group (4.8%). Multiple logistic regression analysis revealed that IHT was an independent predictor of in-hospital mortality (adjusted odds ratio [aOR] 1.114, 95% confidence interval [CI] 1.101–1.128) after adjusting for variables. Sub-analysis indicated that higher severity scores, shorter symptom onset-to-arrival duration, and diagnoses of infectious or respiratory diseases were significantly associated with increased in-hospital mortality among transferred patients. **Conclusions:** This study identifies IHT as a significant factor associated with increased in-hospital mortality. Additionally, it suggested the need for policies to mitigate the risks associated with IHT, particularly in critically ill patients, those with the acute phase response, and those with infectious, genitourinary, and respiratory diseases.

## 1. Introduction

Despite advancements in the emergency medical system, inter-hospital transfer (IHT) remains a crucial component in the emergency medical system. In South Korea, 7.5% of patients visit emergency medical centers through IHT [1]. Various factors determine the necessity for IHT, including the limitations of the initial medical facility in providing the required treatment, the availability of medical equipment and personnel, and patients’ conditions and needs. While transferring a patient is necessary to provide optimal care, it can also introduce risks such as delays, deterioration of the patient’s condition, and errors in the transmission of medical information, all of which can negatively affect patient outcomes.

Several studies have evaluated the impact of IHT on patient outcomes. Some studies have reported positive effects, indicating that transfers can improve patient prognosis when properly managed. Westfall et al. [2] reported that transferred patients had lower mortality than non-transferred patients in patients with acute myocardial infarction. However, other studies have suggested that transfers may worsen outcomes. Muller et al. [3] reported that patients with IHT incur higher medical costs and longer hospital stays than direct visits. Moreover, certain disease groups (acute myocardial infarction, sepsis, stroke, and respiratory disease) have shown higher mortality rates among transferred patients. Faine et al. [4] reported that patients with sepsis who were transferred had higher mortality rates and medical costs compared to those who were admitted directly.

This study aimed to investigate whether IHT is associated with in-hospital mortality in patients admitted to hospitals after visiting the ED and provide foundational data for establishing a more effective transfer system and delivering patient-centered healthcare services.

## 2. Materials and Methods

### 2.1. Study Design and Data Source

This study used data obtained from the National Emergency Department Information System (NEDIS) in Korea. Demographic data, clinical data, and diagnoses of all patients who visited the ED were obtained. This retrospective observational study, based on a nationwide observational cohort, was approved by the institutional review board (IRB) (no. 2021AN0184). The requirement for information was waived due to the retrospective nature of this study.

The collected data included the date and time of ED visit; age; sex; insurance type; onset to arrival duration; route of visit; symptoms; mental status at ED visit; vital signs at the ED visit (oxygen saturation, respiratory rate, pulse rate, blood pressure, and body temperature); triage score (Korean Triage and Acuity Scale [KTAS]: KTAS 1, high severity; KTAS 5, low severity); length of stay (LOS) in the ED; disposition such as transfer, admission, or discharge; diagnosis code; length of hospital stay; and hospitalization outcomes.

### 2.2. Selection of Study Patients

This study included patients aged ≥18 who visited the emergency medical center of a local or regional emergency medical center between January 2016 and December 2018. We did not target all patients who visited the ED but specifically focused on those admitted to the hospital via the ED. Since it was impossible to track the outcomes of patients transferred out after visiting the ED, we focused on patients admitted via the ED for whom the transfer route and post-admission outcomes could be determined. Therefore, this study focused on patients admitted to the hospital via the ED. In Korea, EDs are classified as regional emergency medical centers (level 1), local emergency medical centers (level 2), and emergency medical institutions (level 3). As the NEDIS does not collect emergency medical information from emergency medical institutions, these institutions were excluded from this study. In addition, patients for whom the route to the ED, diagnosis, vital signs, symptom onset-to-arrival duration, or triage were not recorded were excluded.

The study population was divided into a group of patients who were transferred from other medical institutions (transfer group) and a group of non-transferred patients who visited directly through volunteers or outpatients (non-transfer group).

### 2.3. Data Analysis and Outcome

Basic characteristics and initial vital signs were compared between the transfer and non-transfer groups. The primary outcome was in-hospital mortality. Multiple logistic regression analysis was performed to assess the association between IHT and in-hospital mortality after adjusting for selected variables. The modified early warning score (MEWS) was used to adjust for severity. It was calculated based on mental status and vital signs at the time of the ED visit, including oxygen saturation, respiratory rate, pulse rate, blood pressure, and body temperature [5].

Sub-analyses were performed using multiple regression analysis, classifying the severity using the MEWS, diagnosis, and symptom onset-to-ED arrival duration. Patients’ diagnoses were categorized based on the International Classification of Diseases (ICD)-10 by the World Health Organization (WHO). Diagnoses were classified into 22 diagnostic groups according to the diagnosis codes. A sub-analysis was conducted on the seven diagnostic groups with the largest number of patients out of the 22 diagnostic groups. The symptom onset-to-ED arrival duration was defined as “onset time”, and the interquartile range (IQR) of onset time was categorized.

### 2.4. Statistical Analysis

The demographics and characteristics of the study patients were summarized using descriptive statistics. Values are presented as mean ± standard deviation or median (interquartile ranges) for continuous variables and as number (%) for categorical variables. Continuous variables were compared using the unpaired Student’s *t*-test or analysis of variance. Multiple logistic regression analysis was used to assess the associations between IHT and in-hospital mortality after adjusting for selected covariates. Multiple regression analysis was performed to determine the cause of cardiac arrest. All statistical analyses were performed using IBM SPSS version 22.0 (IBM Corp., Armonk, NY, USA). Two-tailed *p*-values < 0.05 were considered significant.

## 3. Results

### 3.1. Study Population and Outcomes

**Figure 1:**
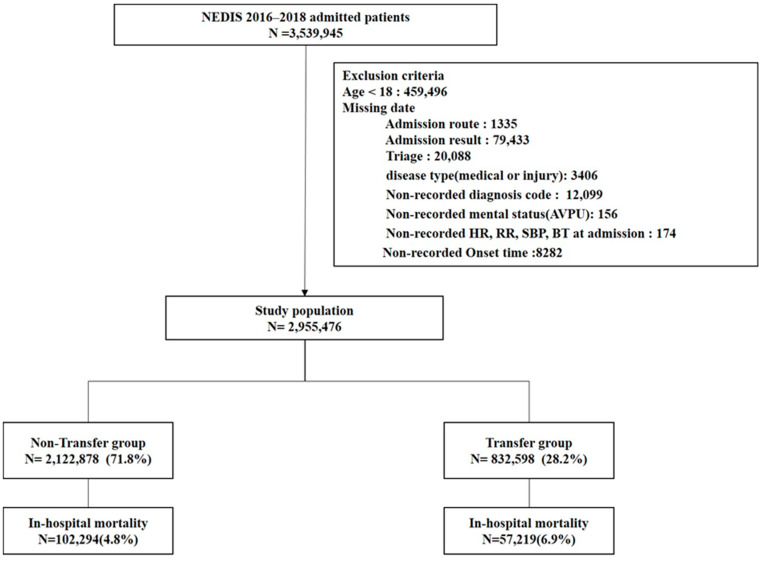
Flowchart of the selection of the study population. NEDIS, National Emergency Data Information System; AVPU, alert verbal pain unresponsive; HR, heart rate; RR, respiratory rate; SBP, systolic blood pressure; BT, body temperature.

Between 2016 and 2018, 27,433,808 patients visited the ED, of whom 3,539,945 were admitted to a regional or local emergency medical center. Of the 3,539,945 patients, the study was conducted on 2,955,476 patients, excluding 459,496 patients under the age of 18 and 124,973 patients with unknown variables. Among the study patients, 832,598 (28.2%) were transferred from other medical institutions, and 2,122,878 (71.8%) patients visited directly. The in-hospital mortality rates in the transfer and non-transfer groups were 6.9% and 4.8%, respectively (*p* < 0.001) (Figure 1).

### 3.2. Comparison of the Basic Characteristics According to Study Groups (Transfer Group vs. Non-Transfer Group) and Outcome (Survivor Group and Non-Survivor Group)

Table 1 presents the characteristics of patients in the transfer and non-transfer groups. Patients in the transfer group were older; the proportion of medical aid in the transfer group was 9.1%, which was higher than that of the non-transfer group (8.6%). The proportion of patients who visited the ED because of injury was 15.8% in the transfer group, which was lower than that in the non-transfer group (19.2%). The transfer group had a higher severity level according to the KTAS classification, a higher ICU admission rate, and a longer duration of hospitalization.

Table 2 shows the characteristics of patients in the survivor and non-survivor group. Compared with the survivor group, the non-survivor group had a higher proportion of male patients and older individuals. Additionally, the non-survivor group had a higher rate of IHT and a longer duration of hospitalization. The IHT rates were 35.9% and 27.7% in the survivor and survivor groups, respectively.

The mortality rates by triage category were as follows: KTAS 1–31.7% (transfer group) vs. 31.0% (non-transfer group); KTAS 2–11.8% vs. 8.8%; KTAS 3–5.3% vs. 4.2%; and KTAS 4 or 5–3.1% vs. 2.2%, respectively.

### 3.3. Multiple Logistic Regression Analysis for In-Hospital Mortality

Multiple logistic regression analysis was performed after adjusting for age, sex, insurance type, disease type, ICU admission, MEWS, onset time, and IHT in patients hospitalized at regional or local emergency medical centers. The adjusted odds ratios (aOR) for in-hospital mortality were 1.442 (95% confidence interval [CI], 1.426–1.458) for male sex, 1.912 (95% CI, 1.874–1.952) for medical disease type, and 1.074 (95% CI, 1.055–1.092) for medical aid. The odds ratio (OR) was 0.684 (95% CI, 0.673–0.696) for an onset time of less than two hours. Multiple logistic regression analysis revealed that IHT (OR, 1.114; 95% CI, 1.101–1.128) was an independent predictor of in-hospital mortality (Table 3).

### 3.4. Sub-Analyses Based on MEWS, ICU Admission, Onset Time, Diagnostic Classification

**Figure 2:**
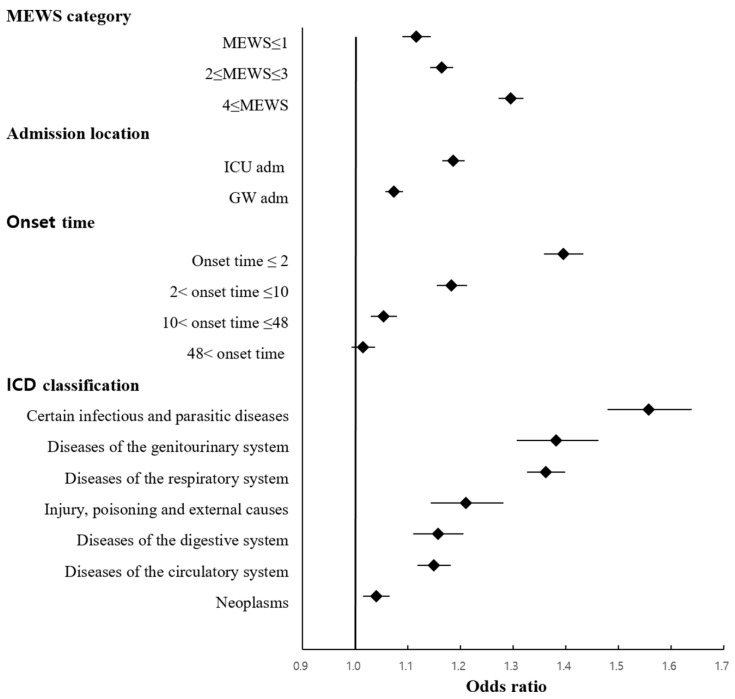
Forest plot of adjusted odds ratios of inter-hospital transfer for in-hospital mortality according to sub-analysis groups after adjusting for age, sex, insurance type, disease type, ICU admission, MEWS, onset time, and IHT. MEWS, Modified Early Warning Score; ICU, intensive care unit; GW, general ward; onset time, interval from the onset of symptom to admission at the emergency department; IHT, inter-hospital transfer; ICD, International Classification of Diseases.

Sub-analyses were conducted to determine the ORs of IHT for in-hospital mortality according to severity classification based on vital signs upon arrival at ED, ICU admission, onset time, and ICD diagnostic classification. Figure 2 shows the ORs of IHT for in-hospital mortality after adjusting for age, sex, insurance type, disease type, ICU admission, MEWS, onset time, and IHT. The ORs of IHT for in-hospital mortality increased with higher MEWS based on vital signs upon ED arrival. Additionally, shorter symptom onset time was associated with higher ORs of IHT. In the patient group with an onset time of more than 48 hours, no significant association was observed between IHT and in-hospital mortality (OR, 1.015; 95% CI, 0.993–1.038). The ORs of IHT were high in patients with infectious and parasitic diseases (OR, 1.557; 95% CI, 1.479–1.639), diseases of the genitourinary system (OR, 1.382; 95% CI, 1.306–1.461), and diseases of the respiratory system (OR, 1.362; 95% CI 1.326–1.399). Additionally, the odds ratios (OR) for in-hospital mortality associated with IHT by triage score classification were as follows: KTAS 1—OR 1.168 (95% CI, 1.124–1.215); KTAS 2—OR 1.149 (95% CI, 1.123–1.175); KTAS 3—OR 1.049 (95% CI, 1.031–1.067); and KTAS 4 or 5—OR 1.109 (95% CI, 1.073–1.146).

## 4. Discussion

In this study, the IHT was associated with in-hospital mortality in patients hospitalized at a regional or local emergency medical center. Since 2003, the NEDIS has been used to collect national emergency medical data in South Korea [1]. This study analyzed nationwide data, offering a broader perspective than previous studies, which primarily focused on diagnosis-specific data.

Our findings align with those of some previous research but contradict others. For instance, several studies have reported lower mortality rates among patients with acute myocardial infarction who underwent IHT than among patients who did not undergo IHT [2,6]. However, Dharma et al. [7] reported that IHT in patients with acute myocardial infarction prolongs the total ischemic time, adversely affecting the outcomes. IHT has been associated with higher mortality in studies on emergency general surgery and spinal epidural abscesses [8,9,10]. However, research on trauma, aortic dissection, and acute kidney injury suggests that IHT does not significantly affect outcomes [11,12,13].

In this study, IHT was identified as a factor associated with in-hospital mortality. IHT may disrupt the continuity of care for emergency patients and introduce communication errors during the transfer process, leading to delays and inaccuracies in the transmission of patient information. This disruption could explain the observed association between IHT and patient outcomes. However, as transfer is sometimes unavoidable during the treatment of emergency patients, it is crucial to identify which patient groups are most likely affected by IHT to mitigate its impact on outcomes.

Muller et al. [3] conducted a study that included hospitalized patients and reported IHT as a factor associated with increased in-hospital mortality in patients with diseases, including congestive heart failure, pneumonia, or urinary tract infection. Similarly, our study showed that among patients classified into the ICD diagnostic groups for infectious diseases, those with diseases of the genitourinary or respiratory system demonstrated a higher association between IHT and in-hospital mortality. Several studies have reported that among patients admitted to ICU, those in the IHT group experienced longer ICU stays and higher mortality rates than those of patients in the non-IHT group [14,15,16]. Our findings also indicated that IHT was significantly associated with in-hospital mortality with unstable initial vital signs and those admitted to ICU. For critically ill patients or those with infectious diseases such as pneumonia and urinary tract infections, the subsequent intensive care following initial treatment is crucial. Therefore, it is hypothesized that IHT (inter-hospital transfer) significantly impacts prognosis in these patient groups. These findings suggest that efforts to reduce IHT should particularly focus on these vulnerable groups.

When developing medical policies, efforts should be made to reduce IHT, especially for critically ill patients and those with acute or infectious conditions. Previous research has suggested that transfers during peak times or at night are associated with poorer outcomes, underlining the need for careful planning and quality management during the transfer process [17,18,19,20,21]. Incorporating these considerations, along with our findings, could enhance patient transfer policies.

This study has several limitations. First, this study has a selection bias, as it did not include all patients visiting the ED but rather focused on those who were admitted to the hospital via the ED, thereby targeting relatively more severe cases. Additionally, the study did not reflect the outcomes of patients transferred out after visiting the ED. Second, the NEDIS data lacks detailed clinical information, such as blood test results, which are essential for calculating severity scores like the Sequential Organ Failure Assessment or Acute Physiology and Chronic Health Evaluation II. Although we used modified early warning scores based on initial vital signs, this may not fully capture the severity of patients’ conditions. Also, due to the retrospective nature of this study and the characteristics of the data, we were unable to include the reasons for transfer, the Charlson Comorbidity Index, or door-to-treatment time. Third, the study did not analyze the medical costs associated with IHT, which is an important aspect of evaluating its overall impact on the healthcare system. Lastly, the study did not account for multiple transfers, as the dataset only indicated whether a transfer had occurred without details on subsequent transfers. This limits our ability to assess the impact of repeated transfers on patient outcomes.

## 5. Conclusions

Analyzing nationwide data from the NEDIS, we identified IHT as a significant factor associated with increased in-hospital mortality. Our study suggests the need for policies to mitigate the risks associated with IHT, particularly for critically ill patients, those with acute phase response, and those with infectious, genitourinary, and respiratory diseases. Moreover, additional research is necessary to further understand and address the complexities of IHT and their impact on patient outcomes.

## Figures and Tables

**Table 1:** The basic characteristics and comparison of transfer and non-transfer groups in patients who were hospitalized at regional or local emergency medical centers.

Variable	Total N = 2,955,476	Transfer Group N = 832,598 (28.2%)	Non-Transfer Group N = 2,122,878 (71.8%)	*p*-Value
Age (year ± SD)	60.92 ± 18.5	62.84 ± 18.5	60.16 ± 18.4	<0.001
Age (year), median (IQR)	63 (48–76)	66 (51–78)	62 (48–75)	<0.001
Sex, male, n (%)	1,574,549 (53.3)	444,651 (53.4)	1,129,898 (53.2)	0.005
Insurance type—medical aid, n (%)	257,892 (8.7)	75,769 (9.1)	182,123 (8.6)	<0.001
Disease type, injury (%)	539,576 (18.3)	131,775 (15.8)	407,801 (19.2)	<0.001
KTAS, n (%)				<0.001
1	74,307 (2.5)	25,984 (3.1)	48,323 (2.3)	
2	454,484 (15.4)	155,089 (18.6)	299,395 (14.1)	
3	1,571,340 (53.2)	477,616 (57.4)	1,093,724 (51.5)	
4	758,818 (25.7)	152,424 (18.3)	606,394 (28.6)	
5	96,527 (3.3)	21,485 (2.6)	75,042 (3.5)	
Classification of emergency medical center, n (%)				<0.001
Regional emergency medical center	1,069,842 (36.2)	378,002 (45.4)	691,840 (32.6)	
Local emergency medical center	1,885,634 (63.8)	454,596 (54.6)	1,431,038 (67.4)	
Modified Early Warning Score—SBP (mmHg), n (%)				<0.001
0 (101 ≤ SBP ≤ 199)	2,504,048 (84.7)	695,080 (83.5)	1,808,968 (85.2)	
1 (81 ≤ SBP ≤ 100)	292,546 (9.9)	92,783 (11.1)	199,763 (9.4)	
2 (71 ≤ SBP ≤ 80 or ≥200)	118,115 (4.0)	33,278 (4.0)	84,837 (4.0)	
3 (SBP < 70)	40,767 (1.4)	11,457 (1.4)	29,310 (1.4)	
Modified Early Warning Score—HR (rate/min), n (%)				<0.001
0 (51 ≤ HR ≤ 100)	2,223,667 (75.2)	629,128 (75.6)	1,594,539 (75.1)	
1 (41 ≤ HR ≤ 50 or 101 ≤ HR ≤ 110)	324,285 (11.0)	96,084 (11.5)	228,201 (10.7)	
2 (111 ≤ HR ≤ 129 or HR < 40)	307,617 (10.4)	81,172 (9.7)	226,445 (10.7)	
3 (HR ≥ 130)	99,907 (3.4)	26,214 (3.1)	73,693 (3.5)	
Modified Early Warning Score—RR (rate/min), n (%)				<0.001
0 (9 ≤ RR ≤ 14)	47,861 (1.6)	16,489 (2.0)	31,372 (1.5)	
1 (15 ≤ RR ≤ 20)	2,417,676 (81.8)	666,434 (80.0)	1,751,242 (82.5)	
2 (21 ≤ RR ≤ 29 or RR < 9)	429,021 (14.5)	133,162 (16.0)	295,859 (13.9)	
3 (RR ≥ 30)	60,918 (2.1)	16,513 (2.0)	44,405 (2.1)	
Modified Early Warning Score—Temperature, T (°C), n (%)				
0 (35 ≤ T ≤ 38.4)	2,753,402 (93.2)	796,036 (95.6)	1,957,366 (92.2)	
2 (T ≥ 38.5 or T < 35)	202,074 (6.8)	36,562 (4.4)	165,512 (7.8)	
Modified Early Warning Score—AVPU score, n (%)				<0.001
Alert (A)	2,696,346 (91.2)	736,775 (88.5)	1,959,571 (92.3)	
Reacting to voice (V)	131,688 (4.5)	47,981 (5.8)	83,707 (3.9)	
Reacting to Pain (P)	99,390 (3.4)	38,293 (4.6)	61,097 (2.9)	
Unresponsive (U)	28,052 (0.9)	9549 (1.1)	18,503 (0.9)	
Onset time (Interval from onset of symptom to admission at ED) (hour), median (IQR), n (%)	10 (2–48)	22 (5–72)	7 (2–38)	<0.001
Onset time (Interval from onset of symptom to admission at ED) (hour, IQR)				<0.001
Onset time ≤ 2	814,951 (27.6)	96,015 (11.5)	718,936 (33.9)	
2 < onset time ≤ 10	693,418 (23.5)	232,577 (27.9)	460,841 (21.7)	
10 < onset time ≤ 48	753,402 (25.5)	243,565 (29.3)	509,837 (24.0)	
48 < onset time	693,705 (23.5)	260,441 (31.3)	433,264 (20.4)	
ED LOS (hour), median (IQR), n (%)	4.17 (2.40–7.73)	4.5 (2.6–8.4)	4.0 (2.4–7.5)	<0.001
Hospital day (day), median (IQR), n (%)	6 (3–13)	7 (3–15)	5 (2–12)	<0.001
ICU admission N, (%)	512,723 (17.3)	191,768 (23.0)	320,955 (15.1)	<0.001
In-hospital mortality N, (%)	159,513 (5.4)	57,219 (6.9)	102,294 (4.8)	<0.001

Abbreviations: SD, standard deviation; IOR, interquartile range; AVPU; alert verbal pain unresponsive; SBP, systolic blood pressure; HR, heart rate; RR, respiratory rate; KTAS, Korean Triage and Acuity Scale; ED, emergency department; LOS, length of stay; ICU, intensive care unit.

**Table 2:** Basic characteristics of patients in the survivors and non-survivors.

Variable	Total N = 2,955,476	Survivor Group N = 2,795,963 (94.6)	Non-Survivor Group N = 159,513 (5.4)	*p*-Value
Age (year ± SD)	62.98 ± 17.8	60.34 ± 18.5	71.09 ± 14.1	<0.001
Age (year), median (IQR)	63 (48–76)	62 (48–76)	74 (62–81)	
Sex, male, n (%)	1,574,549 (53.3)	1,478,706 (52.9)	95,843 (60.1)	<0.001
Insurance type—medical aid, n (%)	257,892 (8.7)	239,608 (8.6)	18,284 (11.5)	<0.001
Disease type, injury, n (%)	539,576 (18.3)	527,411 (18.9)	12,165 (7.6)	<0.001
KTAS, n (%)				<0.001
1	74,307 (2.5)	51,078 (1.8)	23,229 (14.6)	
2	454,484 (15.4)	409,693 (14.7)	44,791 (28.1)	
3	1,571,340 (53.2)	1,500,353 (53.7)	70,987 (44.5)	
4	758,818 (25.7)	741,630 (26.5)	17,188 (10.8)	
5	96,527 (3.3)	93,209 (3.3)	3318 (2.1)	
Classification of emergency medical center, n (%)				<0.001
Regional emergency medical center	1,069,842 (36.2)	1,009,838 (36.1)	60,004 (37.6)	
Local emergency medical center	1,885,634 (63.8)	1,786,125 (63.9)	99,509 (62.4)	
Onset time (Interval from onset of symptom to admission at ED) (hour), median (IQR)	10 (2–48)	10 (2–48)	9 (2–48)	
Onset time (Interval from onset of symptom to admission at ED) (hour, IQR)				<0.001
Onset time ≤ 2	814,951 (27.6)	768,352 (27.5)	46,599 (29.2)	
2 < onset time ≤ 10	693,418 (23.5)	656,960 (23.5)	36,458 (22.9)	
10 < onset time ≤ 48	753,402 (25.5)	716,178 (25.6)	37,224 (23.3)	
48 < onset time	693,705 (23.5)	654,473 (23.4)	39,232 (24.6)	
ED LOS (hour), median (IQR)	4.17 (2.40–7.73)	4.13 (2.40–7.65)	4.75 (2.66–9.27)	<0.001
Hospital day (day), median (IQR)	6 (3–13)	6 (3–12)	8 (2–20)	<0.001
ICU admission, n (%)	512,723 (17.3)	439,224 (15.7)	73,499 (46.1)	<0.001
Inter-hospital transfer, n (%)				<0.001
Yes	832,598 (28.2)	775,379 (27.7)	57,219 (35.9)	
No	2,122,878 (71.8)	2,020,584 (72.3)	102,294 (64.1)	

Abbreviations: SD, standard deviation; IOR, interquartile range; KTAS, Korean Triage and Acuity Scale; ED, emergency department; LOS, length of stay; ICU, intensive care unit.

**Table 3:** Multiple logistic regression analysis for in-hospital mortality in patients hospitalized at regional or local emergency medical centers.

Variable	Unadjusted			Adjusted		
	OR	95%CI	*p*-Value	OR	95%CI	*p*-Value
Age(year)	1.039	1.038–1.039	<0.001	1.034	1.033–1.034	<0.001
Sex, male	1.341	1.327–1.355	<0.001	1.442	1.426–1.458	<0.001
Insurance type-medical aid	1.381	1.359–1.403	<0.001	1.074	1.055–1.092	<0.001
Disease type, n (%)						
Medical	2.816	2.764–2.869	<0.001	1.912	1.874–1.952	<0.001
injury	1.00	-	-			
ICU admission	4.585	4.538–4.633	<0.001	2.154	2.128–2.181	<0.001
Onset time (Interval from onset of symptom to admission at ED) (hour, IQR)						
Onset time ≤ 2	1.012	0.998–1.026	0.098	0.684	0.673–0.696	<0.001
2 < onset time ≤ 10	0.926	0.912–0.939	<0.001	0.753	0.723–0.747	<0.001
10 < onset time ≤ 48	0.867	0.855–0.880	<0.001	0.816	0.803–0.828	<0.001
48 < onset time	1.00	-	-			
Inter-hospital transfer						
Yes	1.458	1.442–1.473	<0.001	1.114	1.101–1.128	<0.001
No	1.00	-	-			

Abbreviations: OR, odds ratio; CI, confidence interval; ED, emergency department; ICU, intensive care unit; IQR, interquartile range.

## Data Availability

The data that support the findings of this study are available from the National Emergency Medical Center under the Ministry of Health and Welfare in Korea, which were used under license for the current study (Number of the Study Data, N20190320311).

## References

[1] Yoo H.H., Ro Y.S., Ko E., Lee J.H., Han S.H., Kim T., Shin T.G., Kim S., Chang H. (2023). Epidemiologic trends of patients who visited nationwide emergency departments: A report from the National Emergency Department Information System (NEDIS) of Korea, 2018–2022. Clin. Exp. Emerg. Med..

[2] Westfall J.M., Kiefe C.I., Weissman N.W., Goudie A., Centor R.M., Williams O.D., Allison J.J. (2008). Does interhospital transfer improve outcome of acute myocardial infarction? A propensity score analysis from the Cardiovascular Cooperative Project. BMC Cardiovasc. Disord..

[3] Mueller S., Zheng J., Orav E.J., Schnipper J.L. (2019). Inter-hospital transfer and patient outcomes: A retrospective cohort study. BMJ Qual. Saf..

[4] Faine B.A., Noack J.M., Wong T., Messerly J.T., Ahmed A., Fuller B.M., Mohr N.M. (2015). Interhospital Transfer Delays Appropriate Treatment for Patients with Severe Sepsis and Septic Shock: A Retrospective Cohort Study. Crit. Care Med..

[5] Subbe C.P., Kruger M., Rutherford P., Gemmel L. (2001). Validation of a modified Early Warning Score in medical admissions. QJM Mon. J. Assoc. Physicians.

[6] Ranasinghe I., Barzi F., Brieger D., Gallagher M. (2015). Long-term mortality following interhospital transfer for acute myocardial infarction. Heart Br. Card. Soc..

[7] Dharma S., Dakota I., Andriantoro H., Firdaus I., Anandira C.P., Radi B. (2023). Interhospital Transfer versus Direct Admission in Patients with Acute ST-Segment Elevation Myocardial Infarction. Int. J. Angiol..

[8] Sakowitz S., Bakhtiyar S.S., Gao Z., Mallick S., Vadlakonda A., Coaston T., Balian J., Chervu N., Benharash P. (2024). Interhospital Transfer for Emergency General Surgery: A Contemporary National Analysis. Am. Surg..

[9] Emanuelson R.D., Brown S.J., Termuhlen P.M. (2022). Interhospital transfer (IHT) in emergency general surgery patients (EGS): A scoping review. Surg. Open Sci..

[10] Pomponio M.K., Khan I.S., Evans L.T., Simmons N.E., Ball P.A., Ryken T.C., Hong J. (2022). Association between interhospital transfer and increased in-hospital mortality in patients with spinal epidural abscesses. Spine J. Off. J. N. Am. Spine Soc..

[11] Hill A.D., Fowler R.A., Nathens A.B. (2011). Impact of interhospital transfer on outcomes for trauma patients: A systematic review. J. Trauma.

[12] Tseng Y.H., Kao C.C., Lin C.C., Chen C.W., Lu M.S., Lu C.H., Huang Y.K. (2019). Does Interhospital Transfer Influence the Outcomes of Patients Receiving Surgery for Acute Type A Aortic Dissection? Type A Aortic Dissection: Is Transfer Hazardous or Beneficial?. Emerg. Med. Int..

[13] Kitchlu A., Shapiro J., Slater J., Brimble K.S., Dirk J.S., Jeyakumar N., Dixon S.N., Garg A.X., Harel Z., Harvey A. (2020). Interhospital Transfer and Outcomes in Patients with AKI: A Population-Based Cohort Study. Kidney360.

[14] Baig S.H., Gorth D.J., Yoo E.J. (2022). Critical Care Utilization and Outcomes of Interhospital Medical Transfers at Lower Risk of Death. J. Intensive Care Med..

[15] Sokol-Hessner L., White A.A., Davis K.F., Herzig S.J., Hohmann S.F. (2016). Interhospital transfer patients discharged by academic hospitalists and general internists: Characteristics and outcomes. J. Hosp. Med..

[16] Durairaj L., Will J.G., Torner J.C., Doebbeling B.N. (2003). Prognostic factors for mortality following interhospital transfers to the medical intensive care unit of a tertiary referral center. Crit. Care Med..

[17] Teng C.Y., Davis B.S., Rosengart M.R., Carley K.M., Kahn J.M. (2021). Assessment of Hospital Characteristics and Interhospital Transfer Patterns of Adults with Emergency General Surgery Conditions. JAMA Netw. Open.

[18] Ofoma U.R., Dahdah J., Kethireddy S., Maeng D., Walkey A.J. (2017). Case Volume-Outcomes Associations Among Patients with Severe Sepsis Who Underwent Interhospital Transfer. Crit. Care Med..

[19] Mueller S.K., Fiskio J., Schnipper J. (2019). Interhospital Transfer: Transfer Processes and Patient Outcomes. J. Hosp. Med..

[20] Murshed I., Gupta A.K., Camilos A.N., Sabab A., Bacchi S., Kovoor J.G., Chan J.C.Y., Maddern G.J. (2023). Surgical interhospital transfer mortality: National analysis. Br. J. Surg..

[21] Nacht J., Macht M., Ginde A.A. (2013). Interhospital transfers from U.S. emergency departments: Implications for resource utilization, patient safety, and regionalization. Acad. Emerg. Med. Off. J. Soc. Acad. Emerg. Med..

